# The Influence of Mental Resilience on the Positive Coping Style of Air Force Soldiers: A Moderation- Mediation Model

**DOI:** 10.3389/fpsyg.2020.00550

**Published:** 2020-04-17

**Authors:** Xiaojun Zhao, Ju Wang, Changxiu Shi

**Affiliations:** School of Education, Hebei University, Baoding, China

**Keywords:** air force soldiers, mental resilience, positive coping style, social support, moderation-mediation model

## Abstract

In this study, 697 air force soldiers from China were investigated with the Chinese Resilience Scale, Military Emotional Regulation Scale, Simple Coping Scale, and Social Support Rating Scale. Structural equation modeling revealed the following: (1) resilience had a positive predictive effect on active coping style; (2) the emotion regulation strategy of self-comfort mediated the relationship between resilience and positive coping style; and (3) social support moderated the latter half of the intermediary process, in which resilience influenced the active coping style of soldiers through self-comfort. The influence of resilience on air force soldiers was a mediating effect, in which resilience could not only directly predict the coping style of soldiers but also influence their coping style through self-comfort. Social support enhanced the influence of self-comfort on coping style. The study has great theoretical and empirical value for promoting the mental health of soldiers and using positive coping strategies.

## Introduction

Given that air force soldiers comprise a high-tech combat team in the transition period of arms in this new era, it is important to compare the willpower, emotional stability, and psychological endurance of air force soldiers using an assessment of military quality. It is also the key to ensuring efficient cooperation among all links in air defense operations. Due to their remote geographical location, strange and closed environment, monotonous life, military training intensity, critical mission requirements, and other special military environmental factors, air force soldiers can easily develop psychological stress responses that cause anxiety, depression, and other uncomfortable emotions, and take a negative response. Coping is the selection and execution of behaviors evaluating the intrinsic or extrinsic requirements. It produces behavioral efforts of an individual to control problems. As an important mediating and regulating factor in the process of psychological stress, coping plays a regulatory role between stress and response results (Folkman et al., 1986) and regulates the mental health of military personnel (Liao, 2014). Studies have shown that a positive coping style is one of the protective factors of mental health (Meng et al., 2011; Yang et al., 2013; Liao, 2014). To improve soldiers’ mental health, attention should be paid to the cultivation of soldiers’ active coping styles and the avoidance of negative coping patterns as much as possible (Johnson et al., 2008).

Coping style refers to the behaviors with which an individual addresses the demands of the internal and external environment in a specific situation. These coping situations are believed to be beyond the ability of an individual and require changing cognitive and behavioral efforts (Ray et al., 1982). Studies on coping styles were previously published and proposed different theories on coping styles (Solomon et al., 1990; Eid, 2003), but most of them focused on the relationship between coping styles and mental health, giving little attention to the influencing factors of military coping styles, especially active coping styles. Positive coping style is a major protective factor for mental health. It is optimistic and positive in problem solving and good at seeking help. According to Ye and Shen (2002), the nature and type of coping styles were influenced by individual personality and situational factors. Some factors of coping styles used by individuals are called coping resources, which can be roughly divided into three parts: physical resources (e.g., health), psychological resources (e.g., personality traits), and environmental resources (e.g., social support). Process theory emphasizes that coping behaviors are tested in specific situations. Coping must be understood in combination with specific stress events and even different stages of event development. Therefore, this study emphasizes the influence of self-traits and social environmental factors on the positive coping style of air force soldiers. We attempt to explore the mechanism of multiple factors on the occurrence of military coping style to promote the use of positive coping styles and provide a basis for improving the mental health of air force soldiers.

Resilience is “the capacity of a system, be it an individual, a forest, a city or an economy, to deal with change and continue to develop.” Resilience is a measure of how well people and societies can adapt to a changed reality and capitalize on the new possibilities offered. Therefore, the resilient system can improve one’s ability to cope with emergencies through self-adjustment to cope with changes. Based on the perspective of positive psychology, a dynamic model of psychological resilience was constructed (Li and Zhang, 2006), which also clearly defined psychological resilience as follows: the internal psychological potential of individuals to successfully adapt by mobilizing all their resources to protect them in the face of setbacks to pursue harmonious self-development. Resilience is a measure of how well people and societies can adapt to a changed reality and capitalize on the new possibilities offered. Resilience is closely related to positive coping styles (Xiao et al., 2005; Zhang et al., 2017). In the daily military environment, psychological resilience training is used to guide soldiers to improve their rational understanding, stimulate their internal potential, and enable them to evaluate and actively respond to situations of stress (Zuo et al., 2013). Study had shown that high self-resilience can allow soldiers who are exposed to the war environment to better integrate into normal life after the war. They have good personal abilities, can withstand stress, and can reduce suicide, alcohol dependence, PTSD, and other problems (Green et al., 2010). Therefore, there are good reasons to believe that resilience has a positive predictive effect on positive coping styles. To guide air force soldiers to use a positive coping style, the research proposes H1 based on previous studies: psychological resilience has a positive predictive effect on soldiers’ positive coping style.

The environment in which the military profession performs its tasks is relatively critical and complex (Miao, 2006). Emotional regulation is the process by which individuals have their own emotions, how they occur, and how they influence the experience and expression of emotions (Gross, 2002). People with high mental resilience recover from negative emotions through both automatic and controlled adjustment according to the theory of double processing of emotions (Tugade, 2010). At the same time, the emotions of combatants are more likely to be affected by the battlefield environment and life factors, resulting in many negative emotions that weaken the overall combat effectiveness of the army. As one of the methods of positive emotion regulation, self-comfort usually refers to individuals in a certain emotional state. By playing down the influence of emotional events on themselves and lowering the requirements of the internal and external environment (i.e., the soldiers’ personal and external environment), they can accept themselves and emotional events to achieve the purpose of influencing their emotional experience (Wang et al., 2009). These states are associated with negative emotions—that we want to end or avoid—and the (quick and effective) restoration of positive emotions/strategies—that we do not want to end and are free to perpetuate (Andringa et al., 2015). On the basis of previous studies, this study posits that self-comfort, the mode of emotional regulation used by soldiers, is a mediating variable worth considering (Hou and Yu, 2006; Wu et al., 2015; Xu et al., 2017). Self-comforting has effectively lowered soldiers’ depression levels (Huang et al., 2015). At the same time, Fredrickson (2004) proposed that positive emotions in negative situations can expand an individual’s instantaneous knowledge-line command system to construct various resources of an individual, including social resources, psychological resources, physical intelligence, and other resources that effectively address the sudden incident. At the same time, the gradual improvement in coping predicts future positive emotions and strengthens the individual’s psychological resilience through this uninterrupted cycle. Peng et al. (2014) and White et al. (2008) found that mental resilience training can effectively improve the level of mental resilience and promote the use of relatively positive emotional regulation methods and the subsequent use of positive coping strategies. In summary, the research proposes H2: self-comfort, a military emotion regulation mode, mediates the relationship between psychological resilience and a positive coping style.

According to the ecological system theory (Krebs, 2009), an ecosystem is a more complex relationship system composed of growing individuals interacting with their surroundings (Liu and Meng, 2009). The relationship between people and the environment is mutual. In this study, from the perspective of the interaction between individuals and the environment, the mediation model to explore the above hypothesis is likely to be regulated by other environmental variables. The research suggests that social support may regulate the relationship between the emotional regulation mode of self-comfort and positive coping. At the same time, positive social relationship is an important factor affecting mental resilience (Bartone et al., 2012). When the emotional regulation mode of self-comfort is carried out, the greater the role of social support is, and the more positive the coping method that will be adopted. Therefore, H3 is proposed: social support regulates psychological resilience and influences the second half of the mediating process of soldiers’ positive coping style through self-comfort.

**FIGURE 1:**
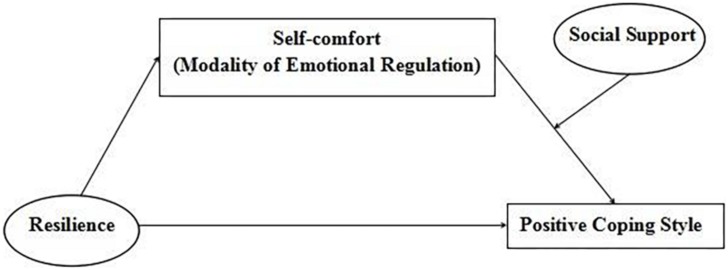
The hypothetical model of the study.

In summary, this study aims to investigate the active coping style of air force soldiers and explores the relationship between psychological resilience and active coping style through a mediated model with moderation (Figure 1). The main purposes of this study are as follows: (i) under the special environment of the army, we probe the relationship between the mental resilience of soldiers and positive coping styles; (ii) we test whether the self-comfort of emotional regulation mediates the relationship between psychological resilience and coping style; and (iii) we examine whether social support has a moderating effect on the mediator. Among these objectives, the first question is to explore the direct connection between resilience and positive coping style, while the other questions focus on the specific mechanism of resilience.

## Materials and Methods

### Participants

A total of 731 questionnaires of soldiers were collected from China, and 34 invalid questionnaires of soldiers were removed. A total of 697 valid questionnaires of soldiers were obtained, with an effective response rate of 95.35%. Among them, the age of the subjects ranged from 17 to 35 years old (*M* = 19.89, SD = 1.86). There are 697 male soldiers with an average military age of 0.96 years.

### Materials

#### Chinese Version of the Mental Resilience Scale

The Chinese version of the CD-RISC mental resilience scale was revised by Yu et al., 2007), which used to measure the level of individual mental resilience. The scale has 25 items that are divided into three dimensions: toughness, self-improvement, and optimism. Each item is scored on a scale from 1 to 5 (1 for “very inconsistent” and 5 for “very consistent”). The higher the score, the higher the level of mental resilience. In this study, the internal consistency coefficient is 0.92, among which the structure of three factors is relatively reasonable.

#### The Scale of Emotional Adjustment of Soldiers

The AERTQ was revised (Wang et al., 2009), adopting a Likert five-point scale. The scale contains 22 questions (including 1 guide question and 1 lie-detection question). The scale covers four dimensions: cognitive attention, emotional appeal, behavioral inhibition, and self-comfort. The alpha coefficient of the scale is 0.769. The internal consistency coefficients of each factor were 0.790, 0.799, 0.667, and 0.731, respectively.

#### Simple Coping Style Scale

The Simple Coping Style Scale (SCSQ), prepared by Xie (1998), was adopted; this scale has a total of 20 items encompassing two dimensions: positive coping (12 items) and negative coping (8 items). The alpha coefficients for the positive coping and negative coping scales in this study were 0.799 and 0.812, respectively, according to a 0–3 score range. The higher the average score of each dimension was, the more frequently it was used.

#### Social Support Rating Scale

The Social Support Rating Scale was revised by Cui et al. (2010), according to the characteristics of the military living and working environment. The scale consists of 10 items encompassing three dimensions: subjective support, objective support, and utilization of support. At the same time, the scale has good reliability and validity. In this study, the internal consistency coefficient was 0.647.

### Procedure

We adopted the method of collective measurement and conducted a group survey with the company as a unit. The main test subjects were trained before the test. During the test, the contents of this study were all approved by the department management and the subjects themselves. At the same time, the principles of anonymous voluntary participation and data confidentiality are emphasized. The amount of time for participants to complete each questionnaire was not to exceed 20 min. The collected data were screened out individually and input manually. We used SPSS 22.0 for the common method deviation test and descriptive statistical analysis of variables. AMOS was used to test the structural equation model.

## Results

### Common Method Bias

Before the statistical analysis of data, Harman’s single-factor test was first used to analyze the common method deviation. Harman single factor test is often used when the source of error cannot be identified. The distinctive approach is to use confirmatory factor analysis while setting a common factor. If this factor accounts for all or most of the variation, a common methodological bias is assumed. Principal component analysis indicated that 18.57% (less than 40%) of the total variation was explained by the first factor; thus, no serious common method deviation problem was observed.

### Descriptive and Preliminary Analysis

The mean, standard deviation, and correlation matrix of the relevant research variables are shown in Table 1. The mean in the table represents the average score of soldiers on each questionnaire. There was a high correlation between the study variables.

**TABLE 1:** Means, standard deviations, and correlation coefficients of variables.

Variable	*M*	SD	1	2	3	4	5	6	7	8	9	10
1. Resilience	3.97	0.54	–									
2. Toughness	3.88	0.58	0.959***	–								
3. Self-improvement	4.24	0.60	0.920***	0.810***	–							
4. Optimistic	3.72	0.64	0.735***	0.602***	0.592***	–						
5. Self-comfort	3.74	0.70	0.483***	0.454***	0.432***	0.405***	–					
6. Social support	3.38	0.57	0.399***	0.374***	0.371***	0.311***	0.322***	–				
7. Objective support	4.67	1.79	0.293***	0.272***	0.276***	0.232***	0.232***	0.860***	–			
8. Subjective support	3.09	0.40	0.294***	0.280***	0.274***	0.214***	0.247***	0.686***	0.286***	–		
9. Support availability	2.88	0.65	0.339***	0.320***	0.303***	0.282***	0.270***	0.593***	0.296***	0.369***	–	
10. Positive coping style	3.14	0.44	0.573***	0.550***	0.493***	0.481***	0.511***	0.442***	0.303***	0.323***	0.439***	–

*p < 0.05; **p < 0.01; ***p < 0.001.

### Influence of Mental Resilience on Coping Style of Soldiers: Mediated Model With Moderation

**FIGURE 2:**
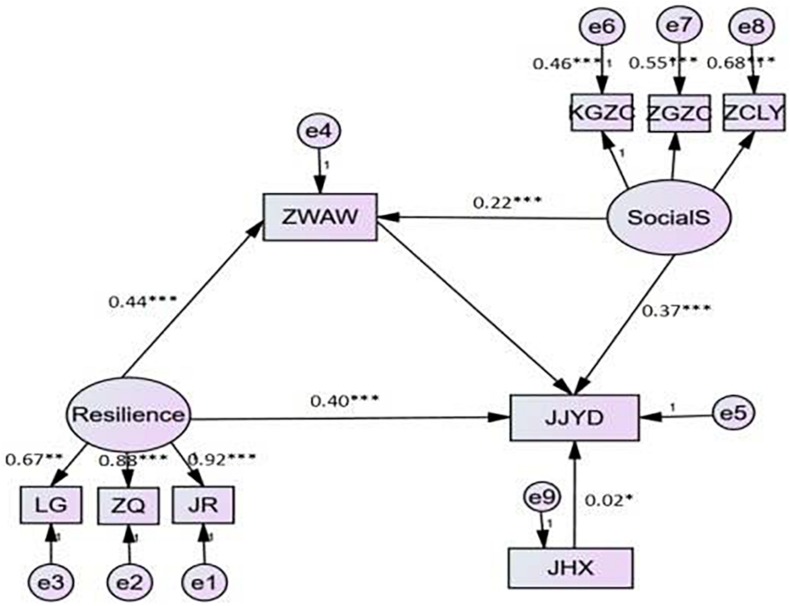
Moderated mediation effect analysis. LG, optimistic; ZQ, self-improvement; JR, tough; JJYD, positive coping style; ZWAW, self-comfort; Social S, social support; KGZC, objective support; ZGZC, subjective support; ZCLY, utilization of social support; JHX, interactive items.

In this study, the maximum likelihood method was used to test the mediated model with moderation by constructing the structural equation model (Wen and Ye, 2014). These models fit the data well—closeness: χ^2^/*df* = 7.67, RMSEA = 0.098, CFI = 0.922, NFI = 0.912, IFI = 0.923. As shown in Figure 2, resilience significantly predicted closeness with positive coping styles: γ = 0.40, *t* = 10.34, *p* < 0.001. Therefore, H1 was supported. Resilience had a positive predictive effect on the emotional regulation of self-comfort: γ = 0.44, *t* = 11.09, *p* < 0.001. Self-comfort significantly positively predicted the positive coping style: γ = 0.21, *t* = 5.62, *p* < 0.001. Therefore, H2 was also verified. Meanwhile, self-comfort × social support significantly predicted positive coping styles: γ = 0.06, *t* = 2.01, *p* < 0.05. Therefore, social support had a moderating effect on the relationship between self-comfort and positive coping style, which is consistent with the H3 statement. In this study, after the addition of mediating variables, such as self-comfort, and moderating variables, such as social support, resilience is also a significant predictor of positive coping style as a dependent variable. Resilience partly mediates positive coping style, and the effect of the moderating variable in the structural equation model, i.e., social support, is also significant. Therefore, the effect of resilience on positive coping style was a mediated model with moderation in this study.

**FIGURE 3:**
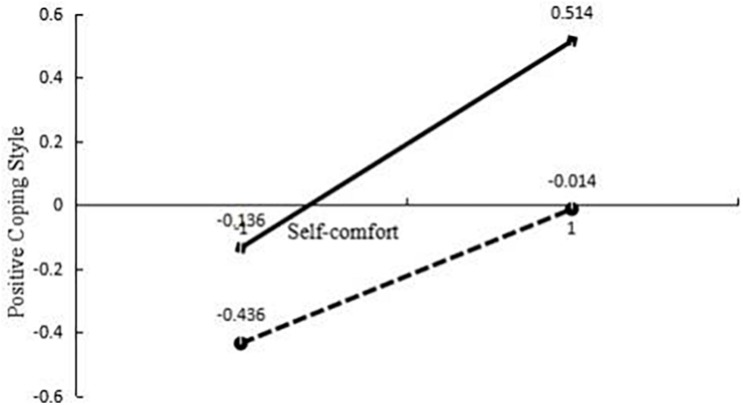
The moderating effect of social support.

In this study, the *z*-score of the moderator variable of social support is ±1, and the interaction effect diagram is drawn. Figure 3 shows how social support regulates the relationship between the mediating variable self-comfort and the positive coping mode. The simple slope test (Widaman, 2006) shows that when individuals have a high degree of social support (the standard score of social support is equal to +1), with an increase in self-comfort, positive coping will show an obvious upward trend (γ = 0.04, *t* = 7.40, *p* < 0.001). For every standard deviation of increase in self-comfort, positive coping increased by 0.33 standard deviation. For air force soldiers with low social support levels, the change in positive coping style is still significant, with an increase in self-comfort levels (γ = 0.05, *t* = 4.64, *p* < 0.001). For every one standard deviation decrease in self-comfort, positive coping increased by only 0.21 standard deviation. In other words, the effect of self-comfort on positive coping styles increased as social support increased.

## Discussion

### Relationship Between Resilience and Positive Coping Style of Air Force Soldiers

Given the professional characteristics of air force soldiers, most of them are in a stressful situation, which is directly related to the efficiency of military training and the completion of military tasks. Therefore, improving soldiers’ psychological regulation and tolerance is one way to improve their mental health. We have carried out a lot of spiritual care research on soldiers who have psychological problems after the war. They incorporate mental health services into military primary care, including centralized workload management; consolidation of psychiatry, psychology, psychiatric nursing, and social work services under integrated behavioral health departments; incorporation of mental health providers into primary care; and routine mental health screening throughout soldiers’ careers (Hoge et al., 2016). Resilience in military environments generally refers to the psychological process in which individuals adapt to or maintain adequate mental health when they are injured (Qiao and Yu, 2013). Therefore, the relationship between mental resilience and coping style is worth exploring. This study found that the stronger the mental resilience of soldiers was, the more frequently they were to use positive coping methods, which was basically consistent with the findings of previous studies (Miao, 2006; Wu et al., 2015). At the same time, soldiers have an environment that is characterized by strict management and high-intensity training. It is particularly important for soldiers to maintain a positive emotion state and to receive support from relatives and colleagues. Therefore, this study focuses on the role of the mediating variable self-comfort and social support, which not only can explain how psychological resilience plays a role in soldiers’ positive coping but also can clarify the specific mechanism of the mediating variable and the moderating variable. This information is of great significance in the study of the mental health of those in the military and the formulation of effective intervention programs.

### Mediating Role of Self-Comfort

The description above verified the relationship between resilience and soldiers’ positive coping style. This study explored the role of the mediating variable self-comfort in this relationship. The results show that psychological resilience significantly predicts self-comfort, which is consistent with previous research conclusions (Xi et al., 2013). The level of psychological resilience can affect the use of self-consolation as an emotional regulation. Levels of psychological resilience can affect the use of self-consolation as a form of emotional regulation. Soldiers with strong mental resilience show more positive emotions. At the same time, they are more willing to use self-comfort as an emotional adjustment and coping method, so that they can be in an optimal emotional state and can adapt better to the environment (Wu et al., 2015). The study also found that self-comfort has a positive predictive effect on the positive coping style. As shown in previous studies, soldiers who tend to adopt the emotional regulation method of self-comfort have higher positive coping levels (Deng et al., 2015). The research results show that specific modes of emotional regulation are an important form of guidance for air force soldiers in their adoption of specific methods of coping.

### The Moderating Effect of Social Support

The second half of the mediating process of social support regulation is discussed using the ecosystem theory of psychological development. This study also further tested whether social support regulates the latter half of the mediating process. As the number of soldiers who used self-comfort as a form of emotional regulation increased, the number of soldiers with more social support who used active coping increased more quickly than those with less social support. For soldiers who are more inclined to use the emotional regulation mode of self-comfort (vs. less self-comfort), soldiers with more social support will use more positive coping methods. Therefore, advocating the use of greater self-comfort as a positive method of emotion regulation can benefit soldiers with more social support. In turn, more social support benefits soldiers who use the relatively positive emotional regulation strategy of self-comfort. In this study, we propose a mediated model with moderation, focusing on the mechanism of independent variables acting on dependent variables, namely, the mediating effect. Second, we consider whether the mediation process is regulated, that is, when the mediating effect is strong and when it is weak (Wen and Ye, 2014). Resilience influences soldiers’ positive coping style through the emotional regulation strategy of self-consolation. The strategy of self-comfort plays a mediating role in the relationship between psychological resilience and soldiers’ positive coping style. With improved social support, the relationship between self-comfort and positive coping strengthens. Based on the perspective of the “ecosystem theory,” in which the social environment influences individual psychological development, there are frequent interactions between individuals and ecosystem factors. This perspective also proves that the influence of social support on individual mental resilience occurs mainly through the influence of individuals’ internal psychological factors and the continuous influence of psychological qualities. Individuals who actually feel a high degree of social support will be willing to develop positive psychological qualities and have more positive resilience (Uchino et al., 1996).

## Future Directions

This study has important reference value for clarifying the internal mechanism of psychological resilience influencing soldiers’ positive coping style. First, we should pay attention to the impact of mental resilience on soldiers’ positive coping styles, conduct psychological intervention as early as possible, and pay attention to improvements in soldiers’ levels of mental resilience. Second, this study found that self-comfort, an emotional regulation style, mediates the relationship between psychological resilience and the positive coping style of soldiers, while social support moderates this mediating process. Therefore, on the one hand, soldiers should be appropriately trained in emotional regulation to improve their ability to regulate their emotions; on the other hand, from the perspective of the social environment, military personnel should be given more social support to create an environment conducive to their safety. This is of great significance in equipping soldiers to use positive coping styles and engage in mental health education.

## Conclusion

In this study, a mediated model with moderation was used to systematically investigate the relationships among mental resilience, self-comfort, social support, and positive coping styles of air force personnel. The research results show that resilience has a significant positive predictive effect on soldiers’ positive coping style. The emotional strategy of self-comfort partly mediated the relationship between resilience and soldiers’ positive coping style. Social support regulated this intermediate process. Compared with individuals who felt less social support, soldiers who felt higher levels of social support were more likely to promote the use of positive coping styles via self-comfort.

## Data Availability Statement

All datasets generated for this study are included in the article/supplementary material.

## Ethics Statement

The studies involving human participants were reviewed and approved by the Academic and Ethics Committee of School of Education in Hebei University. The patients/participants provided their written informed consent to participate in this study.

## Author Contributions

XZ and CS contributed to the conception and design of this manuscript, performed the searches, and conducted the title, abstract screening, and full-text screening. JW performed the data collection and collation. All authors performed the writing of the manuscript and contributed in the revision and approval of the final manuscript.

## Conflict of Interest

The authors declare that the research was conducted in the absence of any commercial or financial relationships that could be construed as a potential conflict of interest.
